# Supplementary material for: Empirical evidence on the impact of the "new round" of Sino-US trade frictions on China’s foreign trade industrial policy and high-quality development
Source: PLoS One. 2024 Oct 2;19(10):e0309458. doi: 10.1371/journal.pone.0309458 (PMC11446427; doi:10.1371/journal.pone.0309458)
Supplement: S1 Appendix — (DOCX) [file pone.0309458.s001.docx]

**Appendix I: Relevant measurement indicators**

**Table 1. Shows the index system of China's foreign trade high-quality development.**

| Primary index | Related words | Symbol | Weight |
| --- | --- | --- | --- |
| Comprehensive trade strength | Total import | - | 0.003283 |
|  | Total export | + | 0.002732 |
|  | Import of goods | - | 0.040229 |
|  | Export of goods | + | 0.018580 |
|  | Service trade export | + | 0.077374 |
|  | Fortune 500 | + | 0020143 |
|  | Export growth rate | + | 0.131329 |
| Innovative development level | R&D expenditure | + | 0.061645 |
|  | Processing trade import | - | 0.069905 |
|  | Processing trade export | + | 0.082719 |
|  | Software export | + | 0.082190 |
|  | Energy saving and environmental protection | + | 0.007264 |
|  | Energy consumption | - | 0.007067 |
| Unimpeded circulation capacity | Cross-border e-commerce | + | 0.022939 |
|  | China-europe freight train | + | 0.031035 |
|  | Highway mileage | + | 0.029705 |
|  | Internet broadband | + | 0.039299 |
| Trade opening and cooperation | pilot free trade zones. | + | 0.019465 |
|  | Contract for foreign projects | + | 0.101317 |
|  | Foreign investment | + | 0.007216 |
|  | Foreign direct investment | + | 0.054360 |
|  | Labor service cooperation with other countries | + | 0.025835 |
| Trade security system | Export duty | - | 0.017523 |
|  | Exchange rate change | - | 0.007800 |
|  | Trade remedy | - | 0.039046 |
