# Supplementary material for: Empirical evidence on the impact of the "new round" of Sino-US trade frictions on China’s foreign trade industrial policy and high-quality development
Source: PLoS One. 2024 Oct 2;19(10):e0309458. doi: 10.1371/journal.pone.0309458 (PMC11446427; doi:10.1371/journal.pone.0309458)
Supplement: Attachment — Submitted filename: response to reviewer 1.docx [file pone.0309458.s002.docx]

Response to Reviewer 1 Comments

Dear Professor,

Thank you for providing your feedback on our manuscript titled "Empirical Evidence on the Impact of the "New Round" of Sino-US Trade Frictions on China's Foreign Trade Industrial Policy and High-quality Development"(MSNumber: PONE-D-24-27007). Your comments are highly valuable to us, and we appreciate thetime and effort you invested in reviewing our work. We carefully considered each comment andmade the necessary revisions, which we hope meet your approval. Please note that we havehighlighted the revised portions in red throughout the paper. In the following section, we summarizethe main corrections made and provide our response to your comments:

***Responds to the Point 1:***

*Point 1. Whether the small values in Table 1 are written as 0 will not affect the result?Please give an explanation.*

Author response to Point 1: The minimum value of table 1 appeared "0", mainly due to for the convenience of showing the results, we take rounding in Table 1, if the fifth is less than “5” will appear the phenomenon of "0.0000", and in the actual process, and in the beginning of the screening process of 1% tail processing, namely there is no value for "0" data, so this does not affect the results of the experiment, of course, in order to avoid differences in understanding, we keep the four decimal behind "0" as "0.0001"

*Point 2.In Table 2 ,why any concerns regarding multicollinearity can be disregarded for subsequent analyses?*

Author response to Point 2: In the correlation analysis, if the coefficient of two variables is between 0 and 0.5, it is considered to have less serious multicollinearity, and if more serious multicollinearity is between 0.5-1. However, the maximum value between the coefficient of these variables is 0.400, that is, the possibility of serious multicollinearity is low in this text. At the same time, multicollinearity is common, slight multicollinearity can be taken without action, that is, reasonable error. In order to avoid the previous statement to be too absolute, we changed the sentence above Table 2, "We can ignore the problem of multicollinearity in the following analysis" to "the maximum correlation coefficient of the main variable is 0.400, that is, the possibility of more serious multicollinearity is small, slight multicollinearity can be taken without action".

*Point 3. In Table 4, there is only a column of the provincial industrial policies, Are the conclusions reliable with one data? Please give a brief explanation.*

Author response to Point 3: The industrial policy selected in this paper is synthesized by financial subsidies and tax incentives through a series of operations (specific formula 19 and 20), table 4 robust test, column (3) and (4) is the fiscal subsidies and tax incentives (16), to avoid misunderstanding, we added four lines in table 4, the column (3) and (4) column variables in *Fs*Treat* and *Tp*Treat* respectively, to avoid the unified use of *Ip*Treat* and misunderstanding.

*Point 4. The following studies were recommended to be properly cited: [1] Spatial-Temporal Ship Pollution Distribution Exploitation and Harbor Environmental Impact Analysis via Large-Scale AIS Data. [2] Orientation-aware ship detection via a rotation feature decoupling supported deep learning approach. [3] Technical and economic analysis of battery electric buses with different charging rates. [4] Manufacturers' Emission-reduction Investments in Competing Supply Chains with Prisoner’s Dilemma: The Economic and Environmental Impacts of Retailer(s) Capital Constraint(s)*

Author response to Point 4: Referring to the appellate literature, we cite it and discuss the inadequacy of this article at the end of the article：However, there are still three shortcomings in this study.first, Limitations to the study data exist, More businesses in 2009 and 2022 and we deleted, at the same time, The enterprise data screened in this paper were largely missing before 2008 and after 2023, After expanding the number of enterprises or the study period, Whether the results remain consistent; next, The choice of the model is too monotonous, The dual difference model is more suitable for the discussion of this paper, But whether the conclusions of the remaining regression models are similarly similar are unclear, For example, the space Dubin model to explore the impact of industrial policies of surrounding provinces on the high-quality development of foreign trade of enterprises in the provinces; last, The validation of the model assumptions cannot be comprehensive, For example, adopting the Levinsohn-Petrin method or the ordinary least squares method to measure the high-quality development of foreign trade and adopting the entropy method to measure the industrial policy level, Making the conclusions more robust. Therefore, the issue of appeal can be further refined in future studies.

Once again, thank you for your comments and we look forward to the formal acceptance of the manuscript.

Yours sincerely,

Fan Sun
